# Multi-omics in HIV: searching insights to understand immunological non-response in PLHIV

**DOI:** 10.3389/fimmu.2023.1228795

**Published:** 2023-08-15

**Authors:** Sonia Espineira, Marina Flores-Piñas, Silvia Chafino, Consuelo Viladés, Eugenia Negredo, Salvador Fernández-Arroyo, Josep Mallolas, Beatriz Villar, Santiago Moreno, Francesc Vidal, Anna Rull, Joaquim Peraire

**Affiliations:** ^1^ Infection and Immunity Research Group (INIM), Institut Investigació Sanitària Pere Virgili (IISPV), Tarragona, Spain; ^2^ Infection and Immunity Research Group (INIM), Hospital Universitari de Tarragona Joan XXIII (HJ23), Tarragona, Spain; ^3^ Universitat Rovira i Virgili (URV), Tarragona, Spain; ^4^ Centro de Investigación Biomédica en Red de Enfermedades Infecciosas (CIBERINFEC), Instituto de Salud Carlos III, Madrid, Spain; ^5^ Lluita contra les Infeccions, Hospital Universitari Germans Trias i Pujol, Badalona, Spain; ^6^ Universitat Autònoma de Barcelona, Barcelona, Spain; ^7^ Universitat de Vic - Universitat Central de Catalunya, Vic, Spain; ^8^ Eurecat, Centre Tecnològic de Catalunya, Centre for Omic Sciences, Joint Unit Eurecat-Universitat Rovira i Virgili, Unique Scientific and Technical Infrastructure (ICTS), Reus, Spain; ^9^ HIV Unit, Hospital Clínic-Institut d'Investigacions Biomèdiques August Pi i Sunyer (IDIBAPS), University of Barcelona, Barcelona, Spain; ^10^ Department of Infectious Diseases, University Hospital Ramón y Cajal, Instituto Ramón y Cajal de Investigación Sanitaria (IRYCIS), Madrid, Spain; ^11^ Universidad de Alcalá (UAH), Madrid, Spain

**Keywords:** genomics, immunological non-response, metabolomics, proteomics, transcriptomics, PLHIV

## Abstract

Antiretroviral therapy (ART) induces persistent suppression of HIV-1 replication and gradual recovery of T-cell counts, and consequently, morbidity and mortality from HIV-related illnesses have been significantly reduced. However, in approximately 30% of people living with HIV (PLHIV) on ART, CD4^+^ T-cell counts fail to normalize despite ART and complete suppression of HIV viral load, resulting in severe immune dysfunction, which may represent an increased risk of clinical progression to AIDS and non-AIDS events as well as increased mortality. These patients are referred to as “immune inadequate responders”, “immunodiscordant responders” or “immune nonresponders (INR)”. The molecular mechanisms underlying poor CD4^+^ T-cell recovery are still unclear. In this sense, the use of omics sciences has shed light on possible factors involved in the activity and metabolic dysregulation of immune cells during the failure of CD4^+^ T-cell recovery in INR. Moreover, identification of key molecules by omics approaches allows for the proposal of potential biomarkers or therapeutic targets to improve CD4^+^ T-cell recovery and the quality of life of these patients. Hence, this review aimed to summarize the information obtained through different omics concerning the molecular factors and pathways associated with the INR phenotype to better understand the complexity of this immunological status in HIV infection.

## 1 Introduction

Effective lifelong antiretroviral therapy (ART) substantially reduces the incidence of acquired immunodeficiency syndrome (AIDS)-related morbidity and mortality in people living with HIV (PLHIV) and transforms HIV infection into a chronic disease by reducing viraemia to undetectable levels and increasing CD4^+^ T-cell counts in peripheral blood (1). However, approximately 30% of ART-treated PLHIV individuals do not achieve optimal CD4^+^ T-cell reconstitution despite achieving complete suppression of HIV replication. They are termed nonresponders, poor immunological recoverees (PIR), discordant responders or immunological nonresponders (INR) (2). There are no universal criteria for INR definition, but several factors have been traditionally linked to INR phenotype, such as age, male sex, drug consumption, late ART introduction, or cytokine storm. The clinical risk is intrinsically related to the current CD4^+^ T-cell count, and of note, the World Health Organization (WHO) considers PLHIV with CD4^+^ T-cell counts less than 200 cells/µL to be the development of advanced HIV disease (AHD) (2–5). Thus, INR are at higher risk of morbidity and mortality for both AIDS and non-AIDS events compared to HIV-infected individuals who have achieved complete immune reconstitution (6, 7), representing an important barrier to achieving the UNAIDS goals to end the AIDS epidemic by 2030 (6). And although much research has been focused on CD4+T-cell reconstitution on ART, the biological and molecular mechanisms determining whether an individual will become an INR are not well understood.

**Figure 1:**
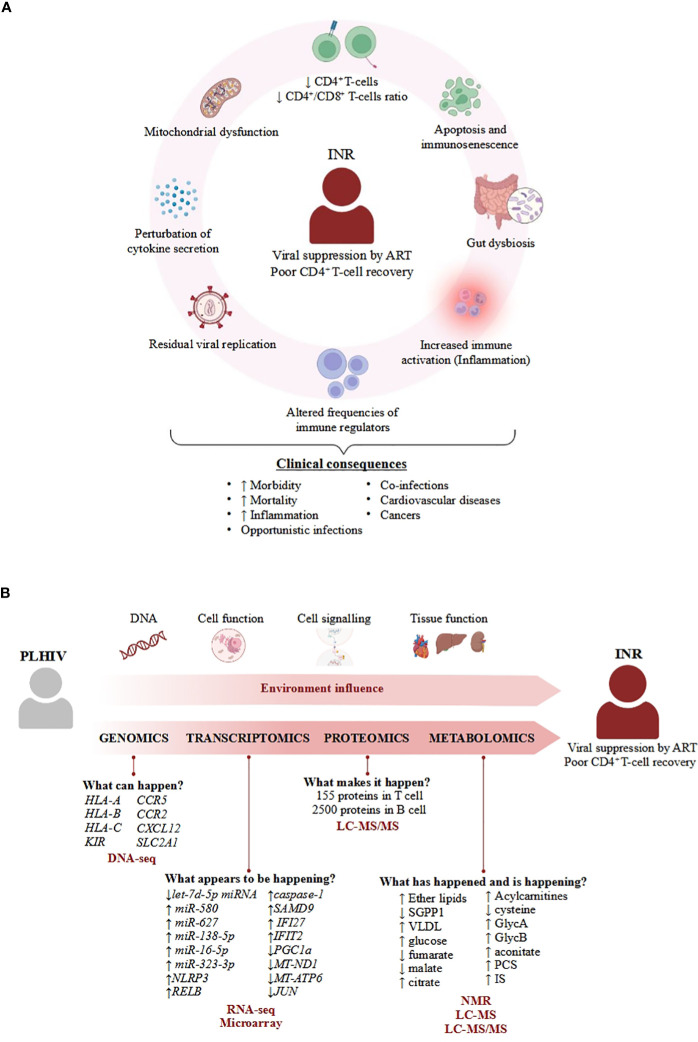
Overview of the physiological and molecular features of INR condition. **(A)** Important processes describing INR that enhance clinical consequences. The mechanisms of the INR condition are characterized by a set of biological processes that eventually lead to different pathologies such as cancers (8, 9), cardiovascular diseases (10), opportunistic infections (11, 12) or other coinfections (13, 14), contributing to the increased mortality and morbidity associated to INR condition. **(B)** Overview of applicability of omics science in INR. Omics can answer biological questions of different layers of knowledge to identify factors that confer a specific environment in the INR phenotype in PLHIV, such as 1) understanding the influence of several SNPs in the specific response to infection; 2) analysing perturbations of cell function under infection; 3) how cell communication activates the immune system; and 4) how the coordination among cells allows tissues to perform their function correctly. The combination of these factors provided by different omics adds complexity to understanding the real causes of poor CD4^+^ T-cell recovery in INR patients, even though these patients achieve viral suppression by antiretroviral therapy (ART). Each molecule associated with the INR phenotype is listed below with the omics approach used to identify it, as well as the different techniques (in red bold) used in the different studies identified in this review.

The underlying mechanisms of the INR condition are complicated and not limited to a low CD4^+^ T-cell count (**Figure 1A**). INR is characterized by severe immune dysfunction that includes malfunctioning and decreased cell production in lymphopoietic tissue, altered frequencies of immune regulators such as regulatory T cells and Th17 cells, residual viral replication, T-cell exhaustion and senescence, perturbation of cytokine secretion, mitochondrial disruption, gut dysbiosis, and specific genetic or metabolic characteristics (7, 15–18). However, none of these independent factors can fully explain the mechanism of INR, which seems to be multifactorial. And in this sense, omics sciences have become a powerful tool for obtaining information in different fields, such as genetics, proteomics, or metabolomics, through reproducible quantification of several molecules in different bio-organic fluids and biological samples. Integration of all these molecules with other immunological, molecular, and biochemical data results in deep knowledge of the behavior of the cells in both physiological and pathological states (19) and allows for the identification of many immunotherapeutic targets for the development of new therapies (20). For this reason, this review aimed to collect the main information obtained in different omics studies carried out on the INR phenotype that will help to advance knowledge on the pathophysiology of poor immunological recovery in PLHIV and to gain insights that will help in the generation of new interventional therapeutic strategies.

## 2 Genomics: the blueprint for an INR phenotype

Genomics refers to the study of the total or part of the genetic sequence information and chemical modifications of an organism, respectively, which includes the identification of key genes and functional elements (21). The use of genomic analysis allows for a better understanding of the molecular mechanisms involved in health interventions and environmental factors in disease, which can be widely used for developing future therapeutic strategies (21). Concretely, different single-nucleotide polymorphisms (SNPs) in genes have been related to some molecular processes, such as T-cell expansion, lymphocyte migration, the cell cycle, survival, and apoptosis, which in turn are associated with the magnitude of CD4**^+^** T-cell count recovery during ART (**Figure 1B**) (22–26).

For instance, several SNPs in human leukocyte antigen (HLA) class I have been associated with lower CD4^+^ T-cell counts. HLA class I is formed by 20 genes classified into A, B, and C subtypes, and their function is to activate immune cells by presenting pathogen-derived peptides present in the membrane of cells to T lymphocytes (27). El-Beeli et al. reported that PLHIV with poor CD4^+^ T-cell recovery, characterized by low levels of nadir CD4^+^ T-cells (<200 cells/ul compared with 300 cells/ul of IR), showed a significantly higher proportion of HLA-A68 and HLA-B15 alleles (**Table 1**) (28). HLA-B13 was correlated with the highest risk of immune failure because of its association with less than 100 cells/ul of nadir CD4^+^ T-cells before ART and with not exceeding 300 cells/ul after 2 years of ART. (**Table 1**) (29). Moreover, while the homozygous HLA-Bw6 epitope promotes CD4^+^ T-cell recovery, the homozygous HLA-Bw4 epitope was associated with poor CD4^+^ T-cell recovery once ART was initiated (**Table 1**) (52). In contrast, the HLA-Bw4 epitope has been associated with suppression of HIV-1 viraemia and the maintenance of a normal CD4^+^ T-cell count in HIV patients not receiving ART. The causes of this different role of the HLA-Bw4 epitope depending on the presence or absence of ART are still unclear (53). Moreover, interaction of HLA class I with killer immunoglobulin-like receptors (KIR), which are present on the surface of natural killer (NK) cells, modulates their activity and is associated with a specific immune response (54). Along this line, the study of Soria et al. investigated the different roles of KIR gene polymorphisms and their HLA class I ligands related to their effect on the immunological response to ART. They postulated that INRs showed higher rates of lower allele frequencies of *KIR2DL3* and *KIR2DS2* than in complete responders. A high frequency of *KIR2DL*3 is related to a lower inhibitory potential and therefore better NK activation, resulting in more efficient control of viral infection. Of note, a low allele frequency of HLA-Cw*04:01 was found in INRs, and hence, the KIR2DS2^+^/HLA-Cw*04:01^+^ multi-allele complex was highly associated with poor recovery of CD4^+^ T-cells (**Table 1**) (30).

**Table 1:** Overview of relevant studies related to INR phenotype.

Author	INR definition	Analytical technique	Interest factors	Conclusions related to INR	Reference
GENOMICS					
El-Beeli et al.	CD4^+^ T-cell count <500 and increase cell count by less than 50-150 cells/mm^3^ per year.	PCR-SSP	*HLA-A68* *HLA-B15*	*HLA-A68* and *HLA-B15* alleles were associated with a poor immune response in HIV patients taking ART.	(28)
Pereira et al.	CD4^+^ T-cell count <500 cells/µL.	PCR-SSO	*HLA-B13* *HLA-B35* *HLA-B39*	*HLA-B13, HLA-B35* and *HLA-B39* alleles are related to the poor immune response during cART.	(29)
Soria et al.	CD4^+^ T-cell count <200/mm under suppressive cART	PCR-SSP	*KIR2DL2* *KIR2DL3* *KIR2DS2* HLA-Cw*04:01	Combination of KIR2DS2^+^/HLA-Cw*04:01^+^ multi-allelic complex was highly associated with poor recovery of CD4^+^ T-cells.	(30)
Carvalho-Silva et al.	PLHIV that gained less than 200 CD4^+^ T-cells/µL during the first year of ART	RFLP	*CCR5Δ32*	Heterozygosity for *CCR5Δ32* and lower CD4^+^ T-cell count before treatment was associated with INR status.	(31)
García et al.	PLHIV starting cART with CD4^+^ T-cells <200 cells/μL	Sequenom’s MassARRAY	*IFN-γ rs2430561* *IL-19 rs2243191*	Polymorphisms in IFN-γ and IL-19 genes significantly impact the probability of not achieving an optimal immune recovery.	(32)
Yeregui et al.	pre-ART low CD4^+^ T nadir <200 cells/μl and CD4^+^ T-cell counts lower than 250 cells/μl at 48 months on ART.	DNA sequencing	*CXCL12 rs1801157* *CCR2 rs1799864_814*	*SDF-1/CXCL12* and *MCP-1/CCL2* prognostic markers of immune failure.	(33)
Restrepo et al.	pre-ART low nadir <200 cells/μl and CD4^+^ T-cell count lower than 200 cells/μl at 48 months on ART	Sequenom’s MassARRAY platform	*CCR2 rs1799864-AG* *CXCL12 rs1801157-TT*	*CCR2 rs1799864-AG* or *CXCL12 rs1801157-TT* genotypes influence the probability of poor CD4^+^ T-cell recovery in the population of HIV patients.	(34)
Masson et al.	CD4^+^ T-cell count of <500 cells/μL despite at least 3 years on cART	DNA sequencing	*SLC2A1 rs1385129* *AKT rs1130214*	The homozygous genotype (GG) associated with S*LC2A1 rs1385129* is linked to poor CD4^+^ T-cell recovery and major frequency of GLUT1 in CD4^+^ T-cells.	(35)
TRANSCRIPTOMICS					
Fu et al.	CD4^+^ T-cell count of less than 350 cells/μl at 24 months on ART.	NGS	let-7d-5p miRNA	620 miRNAs associated with poor immune recovery. ↓ Let-7d-5p miRNA could be a potential biomarker for INRs.	(36)
Lv et al.	CD4^+^ T-cell count 200-500 cells/μL before ART at baseline and increase the CD4^+^ T-cells < 20% over baseline levels after one year.	Transcriptome sequencing by Illumina HiSeq X Ten platform	miR-580 miR-627 miR-138-5p miR-16-5p miR-323-3p	↑ 5 miRNAs predict poor immune response after ART.	(37)
Liu et al.	350 cells/μL of the CD4^+^ T-cells for more than 3.5 years	RNA-seq	216 DEGs	↑ gene expression of 46 genes ↓gene expression of 170 genes	(38)
Younes et al.	CD4^+^ T-cell count below 350/μl for the INRs after 8 years	Microarray	TGF-β signalling IFN-α response OXPHOS related genes	↑ *IFI27, IFIT2* and *SAMD9* *↓ OXPHOS*	(39)
Ghneim et al.	CD4^+^ T-cells <350/µL for the INR after 2 years on TAR	Microarray	3000 DEGs	↓ MYC target genes, *ROS*, *NDUF*s, *COXs* ↑ *NFKB1, NFKB2, RELB*, *NLRP3, IL1B and IL18 INR-B*	(40)
Li et al.	CD4^+^ T-cells <350/µL for the INR after 2 years on TAR	Single cell RNA-seq	1445 genes	↑ *IFIT2, SAMD9* ↓KLRB1, JUN, MT-ND1, MT-ATP6 and *RPS26*	(41)
Woelk et al	CD4^+^ < 200 cells/μl following 48 weeks of HAART	Microarray	40 genes	40 genes differential expressed in PBMCs cell of INR patients.	(42)
PROTEOMICS					
Azzam et al.	CD4^+^ T-cell count of less than 350 cells/μl at 2 years on ART.	LC-MS/MS	155 proteins	153 proteins associated with INR/IR. 99 proteins associated with INR/HC. 97 proteins overlapped between INR/IR and INR/HC.	(43)
Liu et al.	CD4^+^ T-cell count less than 350 cells/μl at 96 weeks on ART.	LC-MS/MS	2500 proteins	2500 proteins TGF-β, NF-κB and CD35, were highly altered in INR patients compared to IR and HC.	(44)
METABOLOMICS					
Scarpellini et al.	PLHIV with fail to increase CD4^+^ T-cell count by at least 30%	Targeted MS/MS	40 acylcarnitines 19 proteinogenic amino acids, ornithine and citrulline, 19 biogenic amines, the sum of hexoses, 76 phosphatidylcholines, 14 lyso-phosphatidylcholines 15 sphingomyelins	↑ Ether lipids ↓ SGPP1 β-oxidation	(45)
Rodríguez-Gallego et. al	pre-ART low nadir (<200 cells/μl) and CD4^+^ T-cell count lower than 250 cells/μl at 36 months on ART (baseline study).	NMR	HDL HDL-Cholesterol HDL- TGs VLDL VLDL-Cholesterol VLDL- TGs LDL/HDL	non-HDL lipoprotein particle ↑ VLDL particles (‘medium’ subclass) ↑ glucose	(46)
Masip et al.	pre-ART low nadir (<200 cells/μl) and CD4^+^ T-cell count lower than 250 cells/μl at 36 months on ART (longitudinal study).	NMR	HDL HDL-Cholesterol HDL- TGs VLDL VLDL-Cholesterol VLDL- TGs LDL/HDL	↑ large HDL-P ↑ small HDL-P (increased from baseline levels, Rodríguez-Gallego et. al)	(47)
Qian et al.	CD4^+^ T-cell count rise after 2 years of < 100 or > 300 cells/μl of ART.	PLC-MS/MS-ESI RP/UPLC-MS/MS-ESI+ RP/UPLC-MS/MS-ESI-	125 lipids 68 amino acids 7 peptides 14 carbohydrates 12 cofactors and vitamins 9 nucleotides 6 energy metabolites	↑ Acylcarnitines (MC, PC, OC, and SC) associated with INR	(48)
Ferrari et al.	CD4^+^ T-cells <350/μL receiving ART for 2 or more years	UPLC-MS/MS	125 metabolites	↑ citrate, aconitate,linolenate ↓ nicotinamide, fumarate, malate and phospholipids ↓ amino acids (isoleucina, alalnina glycine…)	(49)
Nyström et al.	pre-ART low nadir (<200 cells/μl) and rise in CD4^+^ T-cells <50 cells/year in the first 2 years following suppressive ART.	LC-MS technique	200 metabolites	↓ levels of cysteine could be associated with poor CD4+ T-cell recovery	(50)
Malo et al.	CD4^+^ T-cell count rises after 2 years of < 100 or > 300 cells/μl of ART.	NMR	Plasma glycoprotein profiles	↑ levels of GlycA and GlycB associated with a worse immunological state. ↑ levels of baseline glycoprotein concentrations tend to respond less to ART.	(51)

Studies are listed in the order in which they appear in the text. Abbreviations: (INR) immunological nonresponder, (PCR-SSP) sequence-specific primer- polymerase chain reaction, (ART) antiretroviral therapy, (PCR-SSO) polymerase chain reaction-sequence specific oligonucleotide, (RFLP) PCR followed by restriction fragment length polymorphism, (NGS) next-generation sequencing, (DEGs) differentially expressed genes, (LC−MS/MS) liquid chromatography with tandem mass spectrometry, (IR) immunological responder, (HC) healthy control, (MS/MS) targeted quantitative tandem mass spectrometry, (NMR) nuclear magnetic resonance, (UPLC−MS/MS) ultra-performance liquid chromatography–tandem mass spectrometry, (ESI) electrospray ionization, ((RP)/UPLC−MS/MS) reversed-phase chromatography combined with targeted quantitative tandem mass spectrometry and (LC−MS) liquid chromatography–mass spectrometry.

In addition, cytokines, chemokines, and their receptors play a key role in HIV infection by activating the immune system and acting as factors related to HIV pathogenesis (55). For instance, C-C chemokine receptor type 5 (CCR5), which is expressed on the surface of lymphocytes and other cell types, plays a key role in HIV infection due to its major involvement in virus entry and cell-to-cell spread (56). The delta-32 (CCR5Δ32) mutation affecting CCR5 that causes a deletion in the receptor sequence prevents entry of the virus into a cell, and consequently, homozygous delta-32 individuals are substantially resistant to HIV infection (57). PLHIV carrying the heterozygous CCR5Δ32 mutation also had a significantly lower CD4^+^ T-cell count at pre-treatment and insufficient recovery of CD4^+^ T-cell count during ART compared to those homozygous CCR5Δ32 individuals and with those without the mutation (**Table 1**) (31). Therefore, aberrant CCR5 protein blocks the ability to co-stimulate CD4^+^ T-cell activation by inducing T-cell proliferation and differentiation (58, 59). Additionally, several SNPs in genes encoding cytokines and chemokines and their receptors are important in HIV infection and associated with inflammatory INR status, such as *IFNγ rs2430561*, *IL19 rs2243191*, *CXCL12 (SDF-1) rs1801157*, *MCP-1 (*CCR2*) rs1799864_814*, and *fractalkine (CX3CR1) rs373278_814* and *rs3732379*. *CCR2 rs1799864-AG* and *CXCL12 rs1801157-TT* genotypes are considered prognostic markers of poor immunological recovery, whereas the *CX3CR1 249I* polymorphism is related to earlier immune failure (**Table 1**) (32–34, 60).

To activate the immune system, Toll-like receptors (TLRs), a family of type I transmembrane pattern recognition receptors (PRRs), are important due to their capacity to initiate the innate immune response by sensing conserved molecular patterns for early immune recognition of a pathogen or endogenous molecules (61). Specifically, the SNP *TLR9*1635AA has been associated with lower CD4^+^ T-cell counts and higher immune activation of CD4^+^ and CD8^+^ T-cells, which is related to higher plasma levels of interferon-inducible protein 10 (IP10), suggesting a link between the TLR9 polymorphism and inflammation (62). Moreover, not only inflammation but also metabolic disorders are involved in the immune response, as glucose metabolism is important for the proper function of CD4^+^ T cells through the glucose transporter GLUT1 (63). The GLUT1 gene is up-regulated by the activation of the phosphoinositide 3-kinase (PI3K)/protein kinase B (AKT)/mammalian target of rapamycin (mTOR) pathway (PI3K/AKT/mTOR) by IL-7 (64). The up-regulation of the *SLC2A1* gene, which encodes GLUT1, promotes the growth and differentiation of these cells through increasing glucose uptake and the activation of glycolysis (63). In a cohort of PLHIV receiving ART, the polymorphism rs1385129 *SLC2A1* allele, which does not change the amino acid sequence, was significantly associated with lower counts and slower recovery of CD4^+^ T-cells (**Table 1**) (35). In fact, the homozygous rs1385129 SLC2A1 allele and the rs1130214 AKT allele were associated with the major presence of GLUT1 receptor and increased glycolytic activity in CD4^+^ T-cells.

Remarkably, mitochondrial DNA also seems to be involved in the CD4^+^ T-cell count recovery. The mitochondrial haplogroup is defined as a population that shares a similar mtDNA sequence. There are four major haplogroups, HV, U, JT, and IWX, and several minor ones such as H, V, J, and T (65). Slow AIDS progression is associated with haplogroup H and better CD4^+^ T-cell reconstruction, whereas haplogroups J and T are associated with poor CD4^+^ T-cell recovery (66, 67).

In 2019, Greenblatt et al. performed whole-exome genome sequence analysis on 48 women with good CD4^+^ T-cell recovery compared to 42 INR to find exome sequence variations associated with CD4^+^ T-cell recovery. In this study, 41 genes harboring variations were detected using combined multivariate and collapsing (CMC) and kernel-based adaptive collapsing (KBAC) methods. Interestingly, 11 genes associated with CD4^+^ recovery had unknown functions (68), which is evidence that more studies related to genetic function are required to understand their role in patients with INR status.

## 3 Transcriptomics: the regulation of INR blueprint

Transcriptomic studies focus on exploring the expression of the genome. These studies quantify RNA molecules under specific conditions due to the fact that gene profiles are affected by different factors such as the stage of development, physiological and environmental factors, and different pathologies (69).

Many studies in recent years have revealed that, due to their chemical stability and anti-RNase action, endogenous circulating miRNAs are reliable blood biomarkers of disease progression (**Figure 1B**). In this regard, miRNAs play key roles related to the regulation of different viral diseases, including HIV, affecting many developmental pathways and cellular processes (70). Through analysis of the different miRNAs profiles in INR compared to immunological responders (IR) before and after 24 months of viral suppression (VS), researchers identified 299 miRNAs at baseline and 321 after 24 months of VS that were differentially expressed between INR and IR groups. All these miRNAs affect the function of genes involved in several biological processes, such as cell binding and cellular and organelle structure (36). Remarkably, Fu et al. found that expression of the let-7d-5p miRNA was significantly down-regulated in the INR group compared to the IR group, and they proposed the let-7d-5p miRNA as a good biomarker to predict INR condition (**Table 1**) (36). Interestingly, miRNA let-7d-5p is a member of the miRNA let-7 family, which is involved in a wide range of human physiological activities, such as cell development and differentiation and/or regulation of the immune response (71). Specifically, some members of the let-7 family can act as regulators of erythroid differentiation (72). Likewise, erythroid cells that express V-domain Immunoglobulin (Ig) Suppressor of T Cell Activation (VISTA) on their surface promote development of regulatory T cells (Tregs) through cytokine TGF-β production (73). This fact might explain the relation between down-regulation of let-7d-5p miRNA and poor recovery of CD4^+^ T cells in INR.

Another study by Lv et al. identified a panel of five up-regulated miRNAs whose expression negatively correlated with CD4^+^ T-cell count after one year on ART (37). The authors of this study proposed a panel of these five miRNAs (miR-580, miR-627, miR-138-5p, miR-16-5p, and miR-323-3p) as a good biomarker to predict a poor immune response to ART, obtaining a model with 92.9% sensitivity and 91.3% specificity (**Table 1**) (37). Of note, upregulation of miR-627 reduces proliferation of CD4^+^ T-cells through regulation of Ca^2+^ transport, which plays a key role in CD4^+^ T-cell development (37, 74). Also, miR-323-3p can act for itself as a biomarker related to the characteristic failure of immune cell recovery in INR patients (75). Similarly, up-regulation of *NLRP3* and *caspase-1* expression has been associated with immunological nonresponse (76). NLRP3 is a component of the inflammasome complex, and it is essential for activation of the innate immune system against a pathogen (77). In the study of Bandera et al., NLRP3 triggering caspase-1 activation and IL-1β cytokine secretion resulted in inflammatory and pyroptotic cell death, which could explains the failure of immune recovery and the chronic inflammatory state of INR (76).

Liu et al. used RNA-seq analysis to identify 316 differentially expressed genes in PBMCs among INR patients (**Table 1**) (38). A total of 170 genes were down-regulated and 46 were up-regulated in INR compared to IR, and their significant function was related to the type I interferon (IFN) signaling pathway, the mitotic cell cycle process, and the immune response (38). Interestingly, the authors emphasized the function of IFI27, with expression that correlated negatively with CD4^+^ T-cell counts, and obtained a good score as a biomarker to distinguish INR from IR patients, as shown in another study (78). Likewise, Younes et al. found up-regulation of proapoptotic genes *IFI27* and*IFIT2* and down-regulation of *PGC1α* (the master regulator of mitochondrial biogenesis) in cycling CD4^+^ T-cells of INR compared with IR (38). Cycling memory CD4^+^ T-cells are characterized by Ki67 and CD71^+^ expression (markers of cell cycle activation), and their frequency increases despite if total CD4^+^ T-cell number is lower in INR than IR, showing a disruption of CD4^+^ T-cell homeostasis (39). Of note, the down-regulation of *PGC1α* promotes decreased OXPHOS and up-regulation of glycolysis (**Table 1**) (39). Other studies also correlate INR phenotype with mitochondrial dysfunction and activation of apoptosis or senescence in CD4^+^ T-cells. Ghneim et al. classified INR in two subgroups, INR-A and INR-B, based on transcriptomic profile, despite the fact that all these patients showed similar CD4^+^ nadir and count, inflammatory and gut barrier dysfunction markers, age, and years on ART. Concretely, INR-B and IR showed similar transcriptomic profiles, whereas 3000 differentially expressed genes, most of them down-regulated, were found in INR-A. These downregulated genes are involved in cell cycling and metabolism, and for this reason, the authors named this group senescent-INR. Specifically, IRF3 activation, reactive oxygen species (ROS) production regulated by FOXO, TGFβ signaling, inhibition of NF-κB activation, oxidative phosphorylation pathways (*NDUF*s and *COX*s) and down-regulation of proliferation by inhibition of MYC targets were associated with senescent-INR. In contrast, the transcriptomic profile of INR-B showed up-regulation of pro-inflammatory factors such as *NFKB1*, *NFKB2*, and *RELB*, the inflammasome complex (*IL1B* and *IL18*), for which INR-B called Inflammatory-INR (40). Recently, single-cell RNA-seq analysis of PBMCs from two IR and two INR differentially detected 1445 genes in INRs and 1384 in IRs. Specifically, in mucosal-associated invariant T (MAIT) cells, the up-regulation of proapoptotic genes (*IFIT2* and *SAMD9*) and down-regulation of the expression of regulators of T-cell function (*KLRB1* and *JUN*) and mitochondrial biogenesis (*MT-ND1*, *MT-ATP6* and *RPS26*) were observed. In cytotoxic CD4^+^ T-cells, 440 DEGs related to the IFN-γ pathway, leukocyte adhesion, and the regulation of hemopoiesis. In contrast, in CD8^+^ effector T cells were identified DEGs related to antigen processing and presentation and the IFN-γ pathway (41). All these findings suggest that the transcriptional regulation of the senescence/apoptosis program, mitochondrial function, and glycolysis activation are associated with the lack of CD4+ T-cell reconstitution. More work on differential gene expression has been performed to understand poor CD4^+^ T-cell recovery. For example, Woelk et al. found that 40 genes with significantly different expression levels between INR and IR groups had a 10% predictive power to distinguish PLHIV who would experience the ‘good’ or ‘poor’ CD4^+^ T-cell recovery (**Table 1**) (42).

## 4 Proteomics: the means to execute the INR blueprint

Proteomics provides a better understanding of the structure and function of the organism than genomics or transcriptomics. This analysis identifies and quantifies the proteome in a specific cell type or tissue at a precise developmental or cellular stage (79). Several translational modifications and diverse patterns of protein expression in different organs and tissues add complexity when interpreting proteomics results (80) as do internal and external stimuli such as nutritional status, exercise, or diseases that affect proteomic dynamics (81). High-throughput methods to identify proteins include protein array and mass spectrometry (MS) techniques, but because of the higher accuracy and sensitivity of MS measurements and the advanced software used for data analysis, MS is the preferred technique among proteomics approaches (82). The use of proteomics has allowed for the identification and monitoring of biomarkers related to different aspects of HIV infection, including CD4^+^ T-cell reconstitution failure (83). Recent studies provide a comprehensive map of protein interactions associated with INR, which, in turn, facilitates the development of new diagnostic and treatment opportunities (**Figure 1B**).

In 2016, a study used LC−MS/MS on CD4^+^ T-cells from healthy controls (HIV-negative controls) and found an altered T-cell proteome profile in both IR and INR. Azzam et al. identified some deregulated signaling pathways and biological processes, such as translation, cellular stress, eIF2-alpha, mTOR and actin cytoskeleton pathways, in INR. A total of 153 proteins were found to vary significantly between the INR and IR groups, and 99 proteins were significantly altered between the INR and healthy control groups (**Table 1**). These findings showed that the cell cycle and apoptosis were altered in immune recovery failure during ART (43). Moreover, a recent study analyzing the baseline proteome of B cells from patients with acute HIV infection showed approximately 2500 significant proteins differentiated among groups. Specifically, the TGF-β, NF-κB and CD35 proteins were highly altered in INR compared to IR and healthy controls (**Table 1**) (44). The enormous number of altered proteins found in this study suggests that dysregulation of B cells in HIV patients may serve as a predictor of poor CD4^+^ T-cell recovery.

The applicability of high-throughput methods related to INR studies is quite new; hence, in this review, some interesting studies that have employed low-throughput methods, such as ELISA or western blotting, to identify proteins that are potential biomarkers in INR are included. For example, CD5 levels were found to be lower in INR than in IR, which was associated with a lower capacity to respond to immune activation as this protein is a specific antigen of the immune system (84). This study also demonstrated that a combination of low levels of TRANCE and CD5 with high levels of CDCP1, CXCL11, CT5 and SLAMF1 may be used to discriminate INRs from IRs.

Otherwise, the chronic infection and systemic inflammation characteristics of PLHIV result in persistent activation of the immune system, affecting cytokine secretion. Accordingly, it has been shown that IL-2, IL-4, IL-10, IL-17, and IFN-γ levels are significantly lower in INR than in IR (15, 85). These cytokines are strongly involved in T-cell activation and proliferation, and their decrease might lead to their depletion (43, 44, 83). Interestingly, Meyer et al. showed that IL-17 levels and CD4^+^ T-cell counts were negatively correlated with high levels of I-FABP and REG3α in INR individuals compared with IR. I-FABP and REG3α have been described as enterocyte damage markers; hence, mucosal immune dysfunction is a factor that may contribute to the INR condition (86). In addition, previous findings have shown that chronic immune activation and ongoing HIV replication may contribute to the persistence of T-cell dysfunction in patients with poor CD4^+^ T-cell recovery (15, 85). To explore the immunological alterations present before ART initiation, Rosado-Sánchez et al. proposed that elevated IL-6 cytokine levels characterize immune damage in subjects with low CD4^+^ T-cell recovery before ART (87). Therefore, these unfavorable immunologic characteristics may be key to helping clinicians personalize treatment strategies and improve long-term immune recovery outcomes. Other important cytokines that play a key role in immune function in the INR phenotype are adipokines, which are secreted by adipose tissue and produce both proinflammatory and anti-inflammatory effects (88). Because low APLNR and RBP4 concentrations were found to be associated with poor immune recovery, these adipokines were proposed as potential biomarkers of the INR phenotype (89).

## 5 Metabolomics: the real-time processes occurring in INR

Metabolomics is defined as the comprehensive, qualitative, and quantitative study of low-molecular-weight molecules in an organism or biological sample. Unlike genomic and proteomics methods, metabolomics measures endogenous metabolism (metabolomics) as well as perturbations of metabolism caused by environmental factors and pathophysiological processes, offering a snapshot of the physiology of the cell most closely related to the phenotype (90, 91). In general, two types of experimental workflows can be applied in metabolomics: untargeted studies, which are used to identify a wide range of metabolites (profiling) to generate a hypothesis; and targeted studies, which focus on a few specific metabolites when a hypothesis is already postulated. The two most frequently used analytical platforms are liquid (LC) and gas (GC) chromatography coupled to mass spectrometry (MS) and nuclear magnetic resonance (NMR) spectroscopy (**Figure 1B**) (92).

In 2016, Scarpellini B et al. used targeted MS/MS and postulated that the number of ether lipids, as measured by the total acyl-alkyl-containing phosphatidylcholines to total phosphatidylcholines (AGPS) ratio, is up-regulated after one year of follow-up in INRs when a significant decline in sphingosine‐1‐phosphate phosphatase 1 activity (SGPP1, SYNE2 locus) and β-oxidation is also observed (**Table 1**) (45). On the one hand, sphingosine-1-phosphate (S1P) is involved in lymphocyte release into circulation; on the other hand, ether lipids dynamically participate in signal transduction pathways (93, 94). Finally, the authors proposed a combination of five different metabolites and ratios to identify rapid progressors and INR at baseline, with 88.89% sensitivity, 92.31% specificity, 88.89% positive predictive value, and 92.31% negative predictive value (45).

In 2018, NMR analysis revealed high baseline (pre-ART) ratios of non-HDL lipoprotein particles, high levels of VLDL particles, and high concentrations of glucose related to the INR phenotype (46). The positive correlation between medium VLDL particles and ratios of ‘total/HDL particles’ and ‘LDL/HDL particles’ supports the negative link between the ‘LDL/HDL particle’ ratios and CD4^+^ T-cell recovery already reported (**Table 1**) (95). Moreover, the association between plasma glucose levels and low CD4^+^ T-cell counts corroborates previous data, indicating increased glycolytic metabolism in CD4^+^ T-cells because activated immune cells consume glucose at an extremely high rate in PLHIV (96). Of interest, using the same study cohort, the authors evaluated lipoprotein profiles at months 12 and 36 after initiation of ART (47). Both large and small HDL-P concentrations were significantly increased during ART in INR, suggesting that the proportion of small particles and large particles is involved in an atherogenic environment related to cardiovascular risk. Additionally, this follow-up study confirmed the negative correlation between CD4^+^ T-cell counts and medium HDL-P concentrations and corroborated the immunomodulatory role of HDL particles as well as their controversial function in cardiovascular diseases (**Table 1**) (47).

More recently, an untargeted metabolomics experimental design revealed that acylcarnitines, which are fatty acid metabolites synthesized by the combination of carnitine and acyl-CoA (activated fatty acid) and transported into mitochondria, are elevated in INR (48, 97). The authors suggested that an increase in acylcarnitine levels may be related to impaired mitochondrial translocation activity via promotion of β-oxidation, which might induce apoptosis in CD4^+^ T-cells and lead to elevated oxidative stress or membrane disruption (48). Furthermore, regression model combining myristoylcarnitine (MC), palmitoylcarnitine (PC), stearoylcarnitine (SC) and oleylcarnitine (OC) obtained the best discriminatory power to distinguish INRs from IRs with high sensitivity and specificity rates (**Table 1**) (48). In another study, 127 significant metabolites were associated with INR. The citrate and aconitate levels increased, whereas the precursors of NAD (nicotinamide), fumarate, and malate significantly decreased in INR samples compared to IR. Moreover, Ferrari et al. detected alterations in amino acid metabolism, sphingomyelin and phospholipids in the INR group. All these data suggest mitochondrial dysfunction and a reduction in TCA cycle activity. Also, the gut-derived bacterial solutes p-cresol sulfate (PCS) and indoxyl sulfate (IS) were found to increase and be negatively correlated with CD4^+^ T-cell counts. Of note, CD4^+^ T-cells in the presence of PCS and IS stop the cell cycle and down-regulate COXIV and mTFA (mitochondrial proteins), producing inhibition of proliferation and cell death (49). Moreover, Nyström et al. identified more than 200 metabolites associated with HIV recovery using the LC−MS technique (50). They postulated that high levels of cysteine correlate positively with CD4^+^ T-cell numbers and are associated with serine and glycine metabolism, which are important for maintaining redox balance in CD4^+^ T cells (**Table 1**) (50, 98). Overall, the low levels of cysteine in the INR group might be associated with poor CD4^+^ T-cell recovery.

Of interest, glycomics is the subset of metabolomics that aims to identify the structure and function of the complete set of glycans (the glycome) that play a crucial role in modulation of the innate and adaptive immune systems, inflammation, and pathological processes (99). In the study of Malo et al. (51), glycoprotein concentrations in the same study cohort were determined by H-NMR via protein bond N-acetylglucosamine and N-acetylgalactosamine (GlycA) signals and the N-acetylneuraminic acid signal (GlycB) associated with the sugar–protein bond concentration and aggregation state shapes (height and width) (**Table 1**). Additionally, the combination of glycoprotein Groups A and B plus H/W ratios could predict the immunological recovery condition at baseline (51).

## 6 Conclusions

The findings from different omic studies collected in the present review suggest that four main pathways are involved in controlling CD4^+^T-cell fates. Cell cycle, apoptosis and senescence, mitochondrial function, and OXPHOS/Glycolysis are the pathways associated with the poor CD4^+^ T-cell recovery of the INR phenotype. Specifically, several transcriptomic and proteomic analyses claimed that high levels of IFI27 and TGF-β, inhibition of NF-κB and Myc target genes, and actin cytoskeleton down-regulation are associated with low CD4^+^ T-cell count by activation of apoptosis and senescence programs. In addition, the down-regulation of PGC1α, *MT-ND1*, *MT-ATP6*, and *RPS26*, the activation of the PI3K/AKT/mTOR pathway, the high presence of GLUT1 in CD4^+^ T-cell, and alteration in amino acid metabolism, sphingomyelin, and phospholipid levels indicate that TCA activity is reduced by activation of glycolysis instead of OXPHOS producing mitochondrial dysfunction. Mitochondrial dysfunction causes cell exhaustion that reduces the capacity for proliferation and differentiation and the immune response to pathogens, which would explain the poor recovery of the immune cells. Although this review identified several factors and pathways associated with the INR phenotype, it also shows that more integrative studies are required to better understand the causes of poor CD4^+^ T-cell recovery. Indeed, this is one of the great challenges of omics application, i.e., the management and integration of all these data for a correct biological interpretation.

## 7 Limitations and future perspectives

There are no universal criteria for the immunological nonresponse definition. The high disparity in INR criteria makes it difficult to compare data from different studies; thus, standard terms and criteria used by both HIV clinical staff and the research community will be of great interest. Additionally, demographic data such as sex differences, age, or other confounding variables is important for interpreting and integrating different omics data, as phenotype is critically conditioned by environmental influence. Metabolomics (metabolome), which is directly related to phenotype, reflects genetic (genome) and protein (proteome) activity and provides a real-time view of dynamic changes because metabolomics quantifies hundreds of small molecules. Hence, the accuracy of the biological and molecular pathways defined by knowledge of environmental factors is crucial for understanding immunological non-response. Moreover, omics produces a large amount of data that helps investigators identify possible targets, but validation studies are also required to assess the biological function related to developing specific INR therapies. Finally, metabolomics has continually grown, and it has been applied to different sample types where bacterial metabolites may be present. In fact, as suggested through the present review, INR condition seems to be highly related to gut dysbiosis. Nonetheless, microbiome effects on the host metabolome in INR were not included in the present review, as we consider that the microbiome/host metabolism provides a framework for investigation and deserves a more specific review.

## Author contributions

SE, MF-P, SC, AR and JP participated in the conception and design of the review. CV, EN, JM, BV, SM and FV provided scientific discussion. SE, MF-P, SC and AR wrote the manuscript. SF-A, EN, JM, SM, FV and JP revised the manuscript. All authors read and approved the final version of the manuscript.
